# Supplementary material for: Cycloartane-Type Triterpenes and Botanical Origin of Propolis of Stingless Indonesian Bee Tetragonula sapiens
Source: Plants (Basel). 2019 Mar 8;8(3):57. doi: 10.3390/plants8030057 (PMC6473588; doi:10.3390/plants8030057)

Supplemantary Materials

Cycloartane-Type Triterpenes and Propolis Botanical Origin of Stingless Indonesian Bee *Tetragonula sapiens*

Niken Pujirahayu[1,2], Toshisada Suzuki[1] and Takeshi Katayama [1,*]

1 Laboratory of Biomass Chemistry, Faculty of Agriculture, Kagawa University, Kagawa 761-0795, Japan; rahayuken08@gmail.com (NP); (TS)

2 Department of Forestry, Faculty of Forestry and Environmental Sciences, Halu Oleo University, Kendari 93232, Southeast Sulawesi, Indonesia,

* Correspondence: , Tel.: +81-87-891-3083

Figure S1: Scheme of the sucessive fractinations of *T. sapiens* Propolis from Jatibali, South Konawe, Southeast Sulawesi (P1)

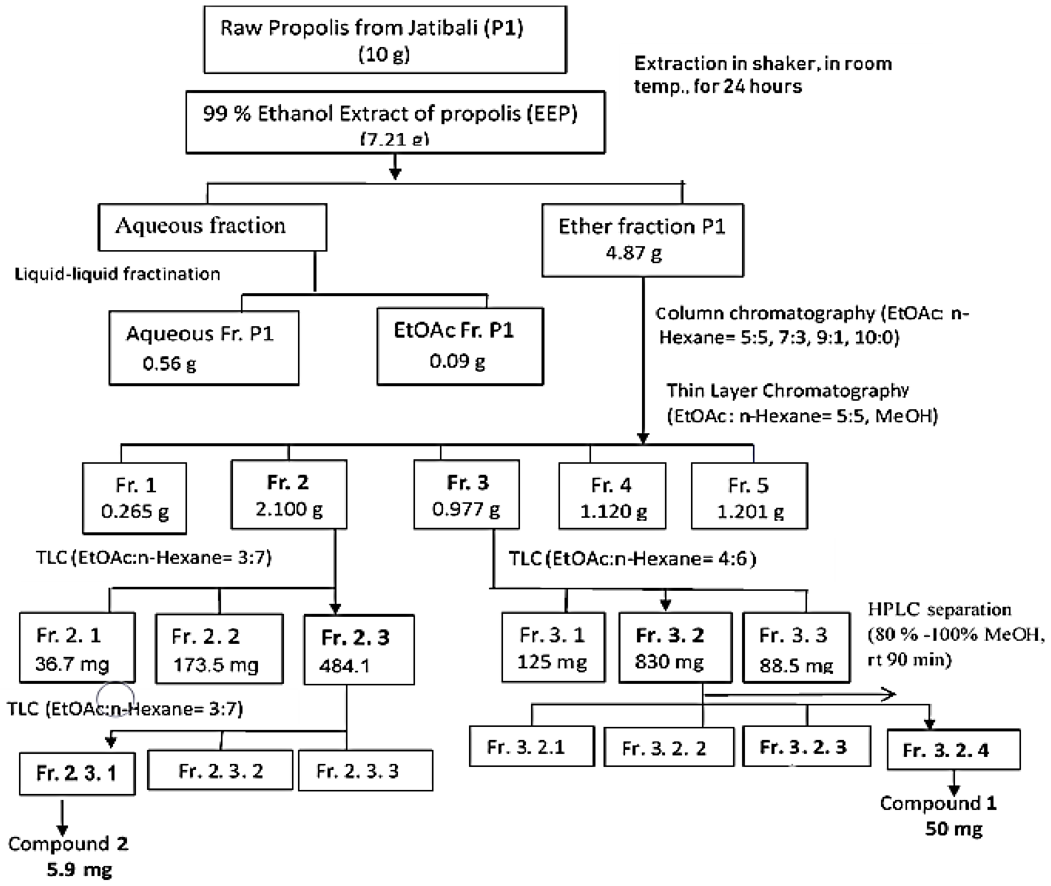

Figure S2: Scheme of the sucessive fractinations of *T. sapiens* Propolis from Kendari, Southeast Sulawesi (P2)

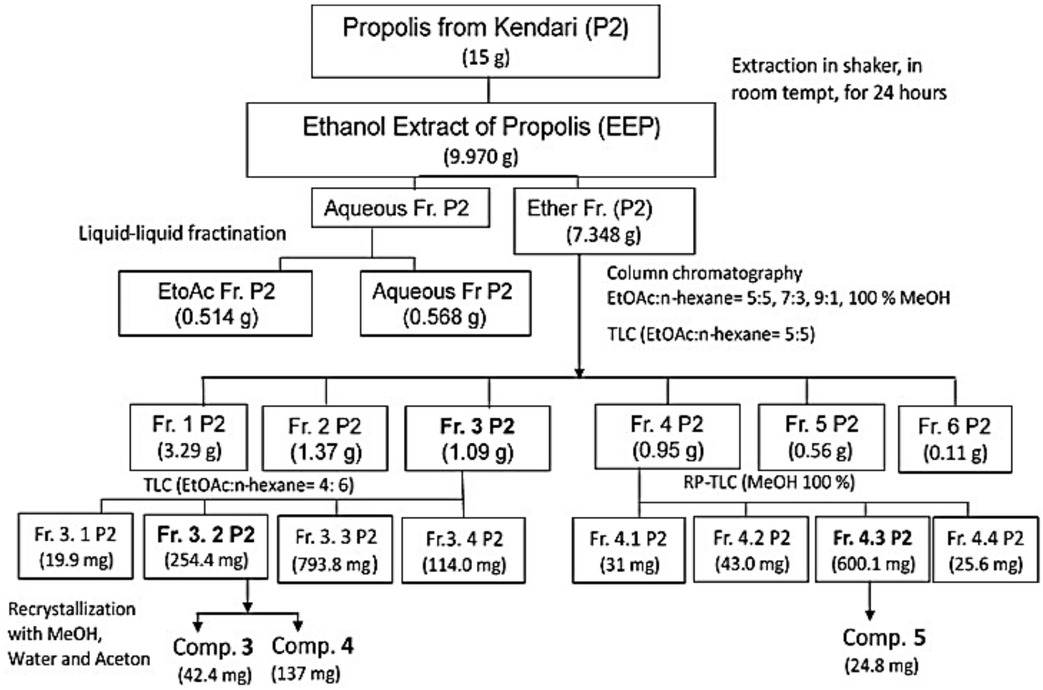

Figure S3: ^{13}C NMR (150 MHz) and ^{1}H NMR spectra (600 MHz, $CDCl_3$) of compound **1 (**mangiferolic acid**)**

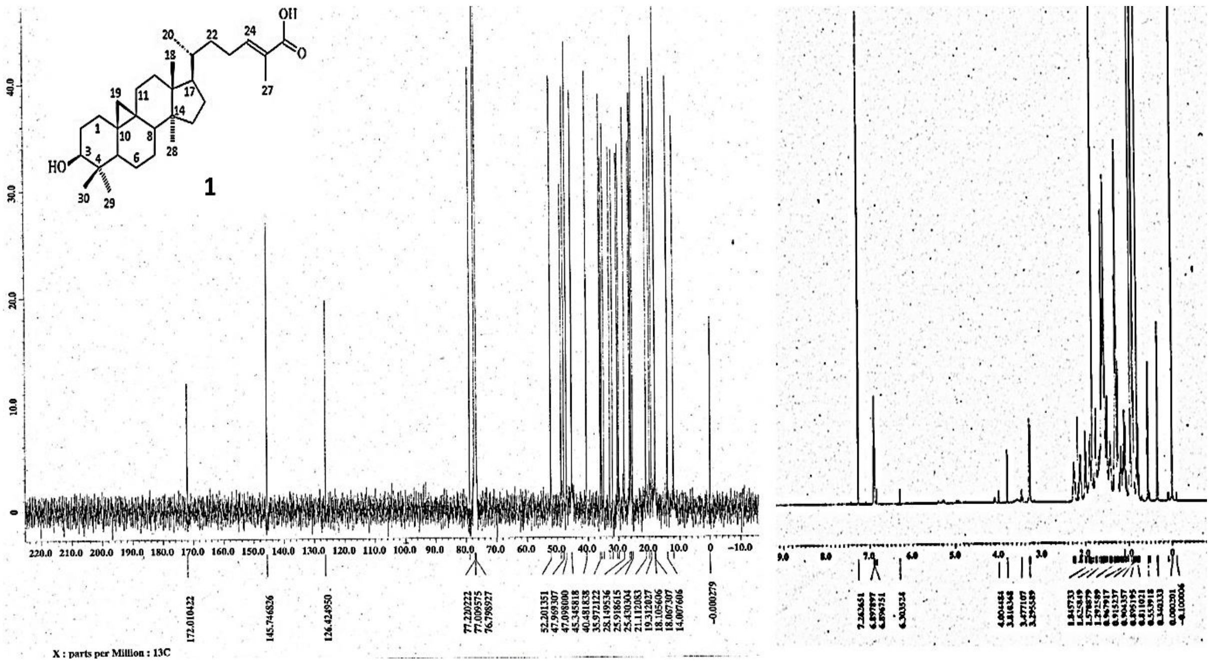

Figure S4: ^{13}C NMR (150 MHz) and ^{1}H NMR spectra (600 MHz, $CDCl_3$) of compound **2** (cycloartenol)

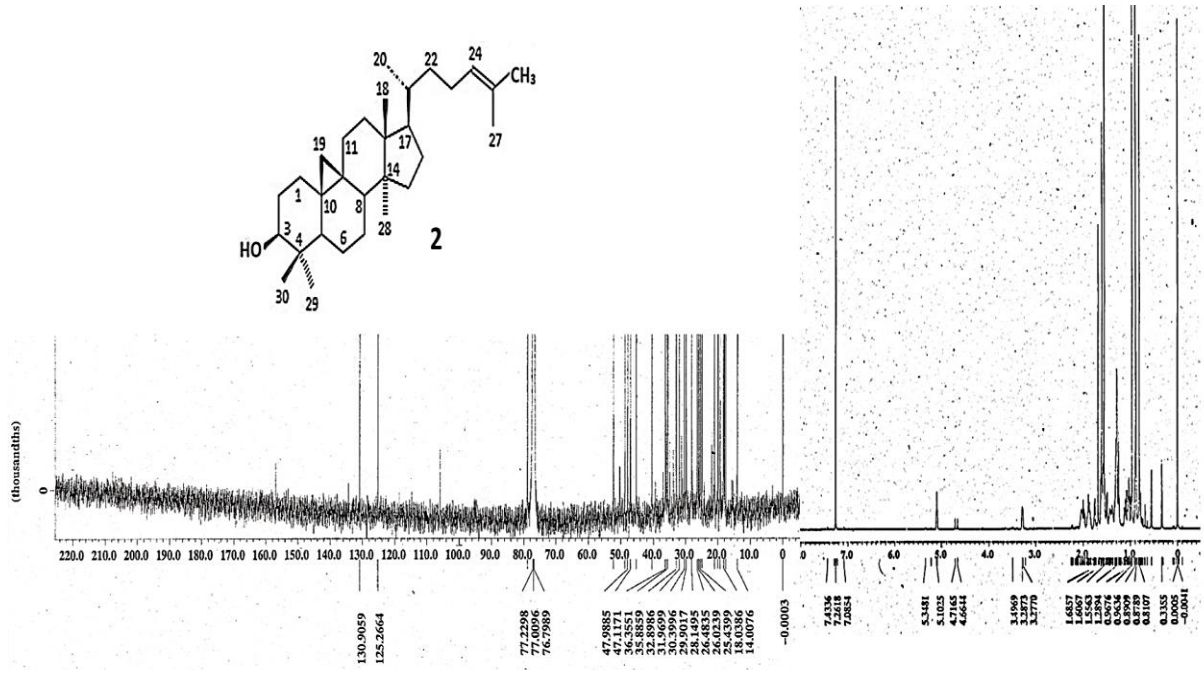

Figure S5: ^{13}C NMR (150 MHz) and ^{1}H NMR spectra (600 MHz, $CDCl_3$) compounds **3** (ambonic acid) and **4** (mangiferonic acid)

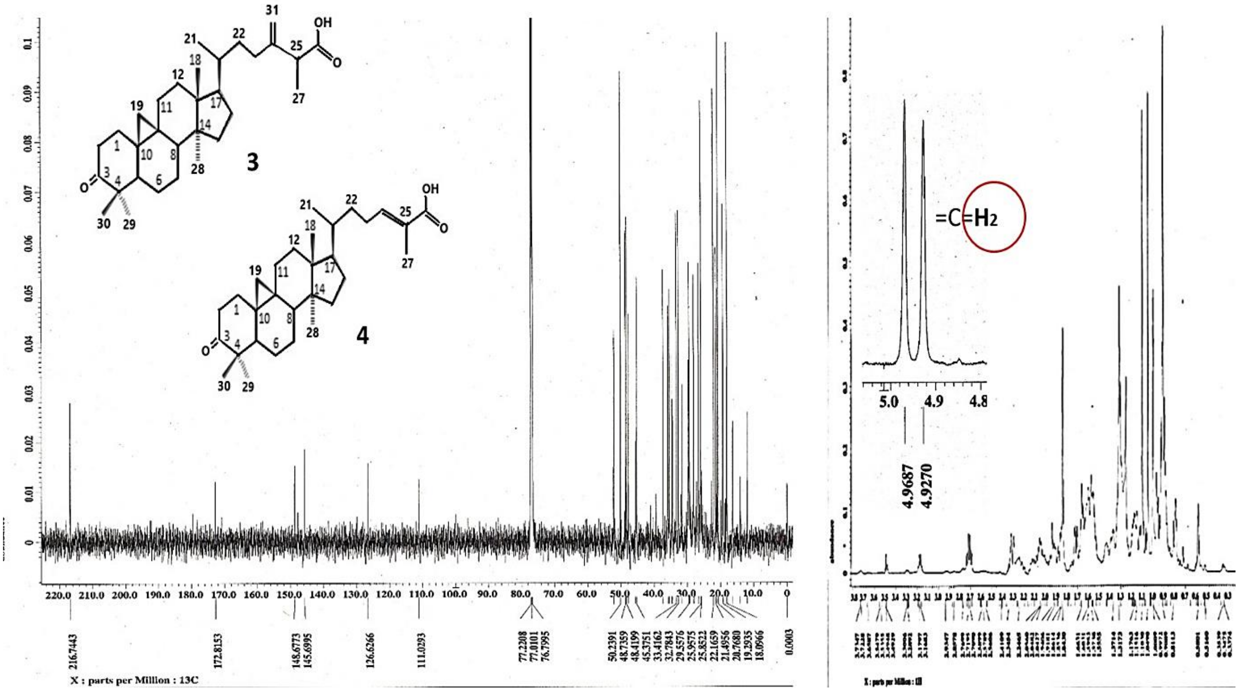

Figure S5: ^{13}C NMR (150 MHz) and ^{1}H NMR spectra (600 MHz, $CDCl_3$) compounds **5** (ambolic acid)

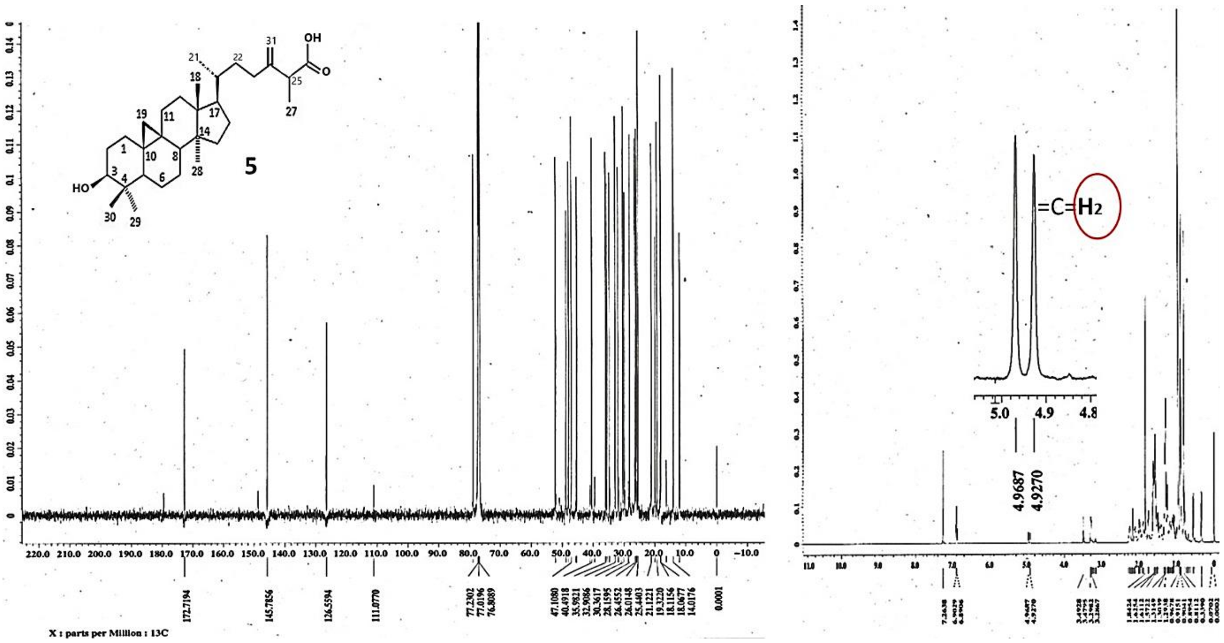

Supplement: Supplementary file 1 [file plants-08-00057-s001.pdf]
